# High PHLPP expression is associated with better prognosis in patients with resected lung adenocarcinoma

**DOI:** 10.1186/s12885-015-1711-1

**Published:** 2015-10-13

**Authors:** Dongqing Lv, Haihua Yang, Wei Wang, Youyou Xie, Wei Hu, Minhua Ye, Xiaofeng Chen

**Affiliations:** 1 Laboratory of Cellular and Molecular Radiation Oncology, Taizhou Hospital, Wenzhou Medical University, Zhejiang Province, 317000 China; 2 Department of Pulmonary Medicine, Taizhou Hospital, Wenzhou Medical University, Zhejiang Province, 317000 China; 3 Department of Radiation Oncology, Taizhou Hospital, Wenzhou Medical University, Zhejiang Province, 317000 China; 4 Department of Thoracic Surgery, Taizhou Hospital, Wenzhou Medical University, Zhejiang Province, 317000 China; 5 Enze Medical Research Center, Taizhou Hospital, Wenzhou Medical University, Zhejiang Province, 317000 China

**Keywords:** PHLPP, Lung cancer, Adenocarcinoma, Immunohistochemistry, Prognosis

## Abstract

**Background:**

PH domain Leucine-rich-repeats protein phosphatase (PHLPP) is a novel family of Ser/Thr protein dephosphatases that play a critical role in maintaining the balance in cell signaling. PHLPP negatively regulates PI3K/Akt and RAF/RAS/′ signaling activation, which is crucial in development, growth, and proliferation of lung cancer. The aim of this study was to investigate the association of PHLPP expression with biological behavior and prognosis of lung adenocarcinoma.

**Methods:**

One hundred and fifty eight patients with pathologically documented stage I, II or IIIA lung adenocarcinoma were recruited in this study. Expression of PHLPP, p-AKT and p-ERK were evaluated by immunohistochemistry (IHC) in paraffin-embedded resected specimens. The correlation of their expression, which was dichotomized to low expression (a score of 0, 1) versus high expression (a score of 2, 3), with the clinicopathological parameters and prognosis of the patients also analyzed.

**Results:**

High PHLPP expression rate in lung adenocarcinoma was 23.4 %. PHLPP expression level was significantly associated with tumor differentiation (*p* = 0.025) and tumor stage (*p* = 0.024). Patients with high expression of PHLPP showed significantly longer average survival time and higher 3 years survival rate than those with low expression of PHLPP (45 months versus 38 months, 85.8 % versus 73.5 % respectively) (Log rank test x^2^ = 7.086, p =0.008). A significant inverse correlation was observed between PHLPP expression and p-AKT (*r* = −0.523, *p* = 0.000) or p-ERK (*r* = −0.530, *p* = 0.000).

**Conclusion:**

Our results suggest that high levels of PHLPP might reflect a less aggressive lung adenocarcinoma phenotype and predict better survival in patients with lung adenocarcinoma. PHLPP can be a potential prognostic marker to screen patients for favorable prognoses.

## Background

PHLPP (PH domain leucine-rich repeats protein phosphatase) represents a family of novel Ser/Thr protein phosphatases. PHLPP has been identified to negatively regulate PI3K/Akt and RAF/RAS/ERK signaling activation [1, 2]. PI3K/Akt pathway plays a central role in inhibiting apoptosis in a variety of cell types including human cancer cells [3]. The RAF/RAS/ERK pathway plays a critical role in numerous cellular processes, including proliferation, differentiation, survival, and motility. Hyperactivation of RAF/RAS/ERK signaling is critical to the development of many human malignancies tumor [2, 4]. The functional importance of PHLPP as a tumor suppressor in different types of cancer has been investigated in several recent studies [1, 2, 5–13]. The expressions of PHLPPs were frequently lost in a variety of human cancers, such as glioma [6], colon cancer [7, 8], prostate cancer [9], gastric cancer [10] and gallbladder cancer [11]. PHLPP expression was significantly associated with progression-free survival in gallbladder cancer [11]. The decrease in PHLPP1 level was highly correlated with shorter survival for patients with pancreatic ductal adenocarcinoma [12]. Patients with low PHLPP1 and PHLPP2 protein expressions have a poor prognosis and PHLPP1 was an independent prognostic factor in hypopharyngeal squamous cell carcinoma [13]. However, the expression and functional significance of PHLPP in lung adenocarcinoma are not clear. The present study aimed to investigate the association of PHLPP expression with biological behavior, clinicopathological characteristics and prognosis of lung adenocarcinoma.

## Methods

### Patients

Clinical data were compiled for 158 patients diagnosed with lung adenocarcinoma in resected specimen from 2008 to 2010 in Taizhou Hospital of Zhejiang Province. Variables included age, sex, date of diagnosis, stage at diagnosis, and time of follow-up. The follow-up was performed every 3 months after surgery for 2 years and once every 6 months thereafter. The median follow-up period was 38 months (range 3–56). Survival curves based on stage at time of diagnosis showed the expected patterns. All patients signed the informed consent. This study was approved by the Ethics Committee of Taizhou Hospital, and tissue specimen acquisition was carried out in accordance with institutional guidelines.

### Therapy

All patients had an Eastern Cooperative Oncology Group (ECOG) perfor-mance status of 0 or 1, adequate baseline organ function defined as a leucocyte count > 4 × 109 (absolute granulocyte count > 2 × 109, platelet count > 100 × 109, normal liver function tests and serum creatinine level < 1.4 mg/dl) and no other severe co-morbid conditions. Patient stage was redetermined according to the TNM Staging System of AJCC (7th version, 2009). Surgery was performed as the initial treatment for pathologically documented stage I, II and IIIA. Postoperative first-line chemotherapy was added depending on the TNM staging system. Cycles were repeated every 3 weeks and four cycles were delivered in II and IIIA patients.

### Immunohistochemistry

At the tissue bank of Taizhou Hospital, tissue arrays were prepared using a Beecher manual arrayer. Five-micrometre-thick sections of the paraffin-embedded tissue blocks were cut and mounted on polylysine coated slides. They were dewaxed in xylene and rehydrated through a graded series of ethanol. After de-paraffinization, antigen retrieval treatment was performed at 120°c for 5 min in a 10 mM sodium citrate buffer (pH 6.0). Endogenous peroxidase activity was blocked by using a 3 % hydrogen peroxide solution at room temperature for 15 min. Then, PHLPP (1:100, ab84978, Abcam, Cambridge, England.), p-AKT (1:100, BS4007, Bioworld Technology, MN,USA.) and p-ERK (BS5016, 1:200, Bioworld Technology, MN,USA.) were applied and incubated overnight at 4°c. After that, a thorough washing in a 0.01 M phosphate-buffered saline (PBS) solution was done. The samples were then incubated with biotin-labeled goat anti-rabbit secondary antibody. Subsequently, binding sites of the primary antibody were visualized using a Dako EnVison kit (Dako, Glostrup, Denmark) according to the manufacturer’s instructions. Finally, sections were counterstained with haematoxylin and mounted with glycerol gelatin. The immunohistochemical specificity of the antibodies was confirmed using two types of negative controls: (i) substituting rabbit non-immune IgG for the primary antibodies, and (ii) omitting the primary antibodies from the staining protocol. Normal colonic mucosa slide was used as a positive control.

### Staining evaluation

Staining in tissues was evaluated by three pathologists who were blinded to any clinical details related to the patients. Membrane staining for PHLPP, cytoplasmic staining for p-AKT and nucleus staining for p-ERK were evaluated. The results of staining were scored according to the intensity of staining with Fourtier system (level 0–3: negative = 0, weakest = 1, moderate = 2, strong = 3) [14]. Then, these scores were divided into PHLPP low expression group (0–1 point) and PHLPP high expression group (2–3 point).

### Statistical analysis

All statistical analyses were conducted using SPSS for Windows (version 17.0). Count data statistics using chi-square test. Associations between continuous variables were analyzed by Pearson’s correlation test. Kaplan-Meier survival curves were calculated for the patient subgroups of interest and compared statistically, censoring for age and stage, using the log-rank test. A *p*-value < 0.05 was considered statistically significant.

## Results

**Fig. 1:**
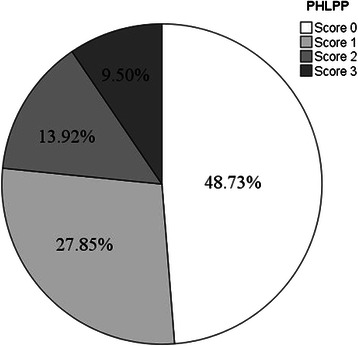
The proportion of patients with different levels of PHLPP expression in lung adenocarcinoma. The structure of cases by the immunostaining scores of PHLPP expression highlights the relatively low percentage (23.4 %) of cases with PHLPP, with score 2 (13.9 %) and 3 (9.5 %). Almost half (48.7 %) from all cases were shown to have no expression of PHLPP

A total of 158 patients with histologically proven lung adenocarcinoma were evaluated in this study. The expression of PHLPP, p-AKT and p-ERK in lung adenocarcinoma were evaluated by immunohistochemistry. The relationship between the expression of PHLPP and the clinicopathological characteristics of lung adenocarcinoma were analyzed. The results showed that the PHLPP expression was associated with histological differentiation and pathological T stage in lung adenocarcinoma (Table 1). Figure 1 showed the proportion of patients with different levels of PHLPP expression in lung adenocarcinoma. The structure of cases by the immunostaining scores of PHLPP expression highlights the relatively low percentage (23.4 %) of cases with PHLPP, with score 2 (13.9 %) and 3 (9.5 %). Almost half (48.7 %) from all cases were shown to have no expression of PHLPP. Patients whose tumors stained at level 2 or greater were designated high expression. Patients whose tumors stained at level 1 or 0 were designated low expression.

**Table 1:** Distribution of PHLPP expression in lung adenocarcinoma lesions according to clinicopathological parameters

	No. of case	PHLPP expression				p*
		0	1	2	3	
Age (Median = 59 years)						
≤ 59 years	80	43	20	11	6	0.515
> 59 years	78	34	24	11	9	
Gender						
Male	42	21	9	8	4	0.357
Female	116	56	35	14	11	
Differentiation						
Well	24	2	12	7	3	0.025
Moderately	93	46	25	12	10	
poorly	41	29	7	3	2	
T stage						
T1	25	12	3	6	4	0.024
T2	97	43	38	14	2	
T3	36	22	3	2	9	
N stage						
N0	69	21	29	12	7	0.322
N1	40	26	8	4	2	
N2	49	30	7	6	6	
Stage						
I	51	9	29	10	3	0.896
II	51	31	9	6	5	
III	56	37	6	6	7	

*p is PHLPP difference between high and low expression. High expression is 2 and 3, and lower expression is 0 and 1

**Fig. 2:**
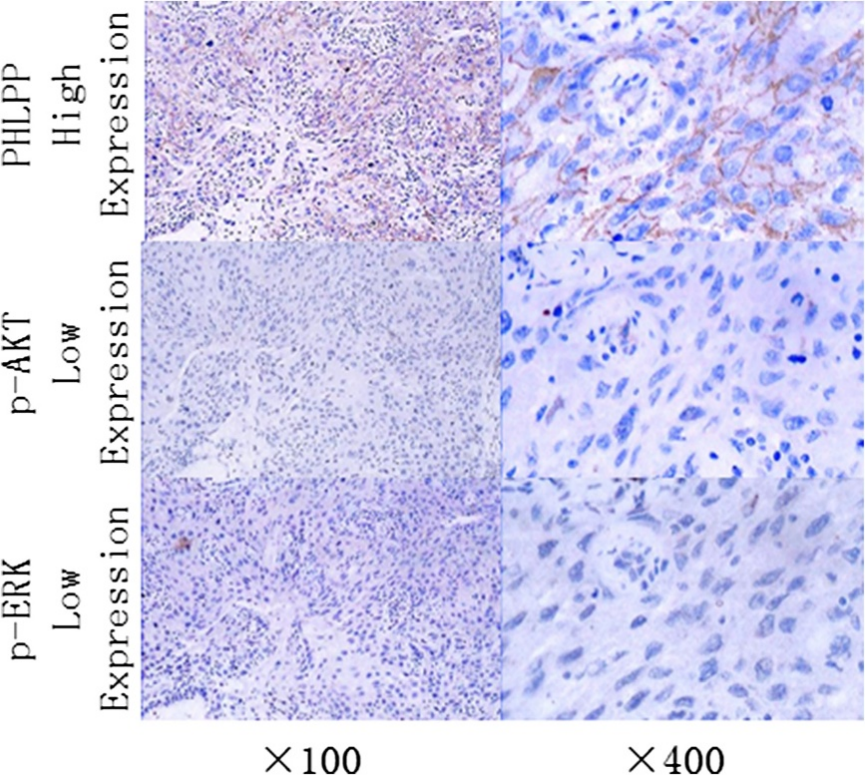
Representative images of immunostaining of PHLPP, p-Akt and p-ERK expression in lung adenocarcinoma. The tissue sections from the lung adenocarcinoma were stained with the PHLPP (upper panels), p-AKT (middle panels) or p-ERK (lower panels) antibodies. The entire section was assessed at low (100x) (left panels) and high (400x) power (right panels) magnification

Representative images of immunostaining of PHLPP, p-Akt and p-ERK expression in lung adenocarcinoma are shown in Fig. 2. There were 77.22 % (122/158) of tumor tissues that had lost PHLPP expression. Among those with loss of PHLPP expression tumor tissues, the p-ERK positive rate was 63.11 % (77/122), p-AKT positive rate was 62.30 % (76/122), p-ERK and p-AKT both positive rate was 25.41 % (31/122) (Table 2). A significant negative correlation was observed between PHLPP expression and p-AKT (*r* = −0.523, *P* = 0.000) or p-ERK (*r* = −0.530, *P* = 0.000) (Table 2).

**Table 2:** The correlation between PHLPP, p-AKT, p-ERK for lung adenocarcinoma

		Pearson coefficient(γ)	*p* value
p-AKT	PHLPP	−0.52	0.000
p-ERK	PHLPP	−0.53	0.000
p-AKT	p-ERK	−0.15	0.055

**Fig. 3:**
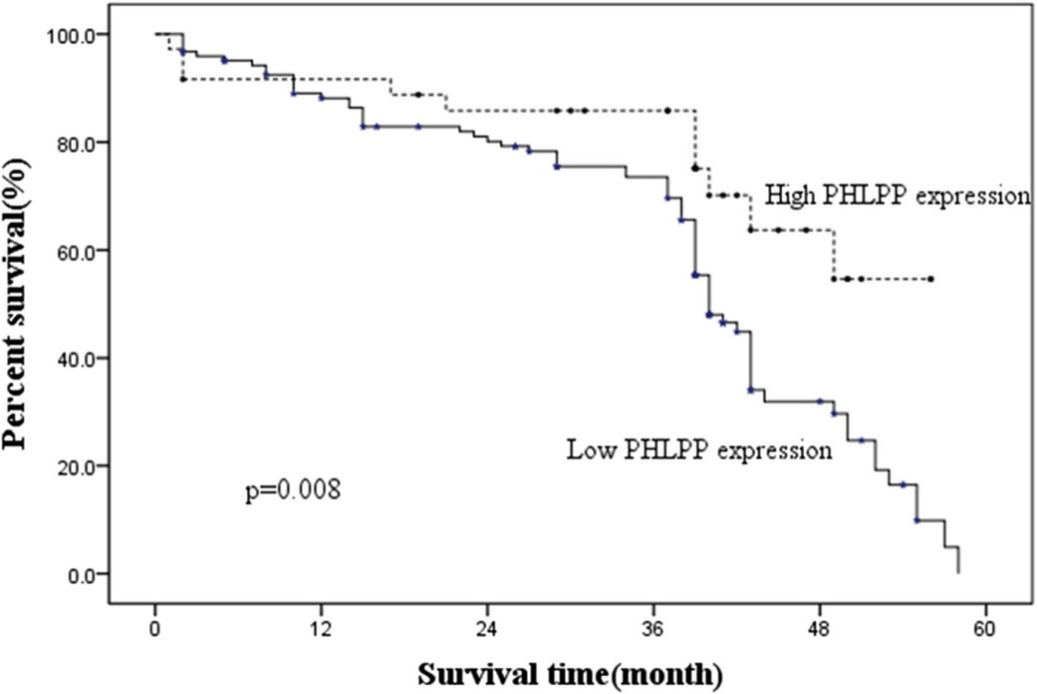
Kaplan-Meier survival curves of patients with high and low expression of PHLPP. Patients with high expression of PHLPP showed significantly longer average survival time and higher 3 years survival rate than those with low expression of PHLPP (Log rank test x^2^ = 7.086, *P* =0.008)

Among the 158 cases, there were 36 cases in which lung adenocarcinoma tissue demonstrated high expression with PHLPP antibody and 122 cases with low expression of PHLPP. Patients with high expression of PHLPP showed significantly longer average survival time and higher 3 years survival rate than those with low expression of PHLPP (38 months versus 45 months, 73.5 % versus 85.8 % respectively) (Log rank test X2 = 7.086, P =0.008) (Fig. 3).

**Fig. 4:**
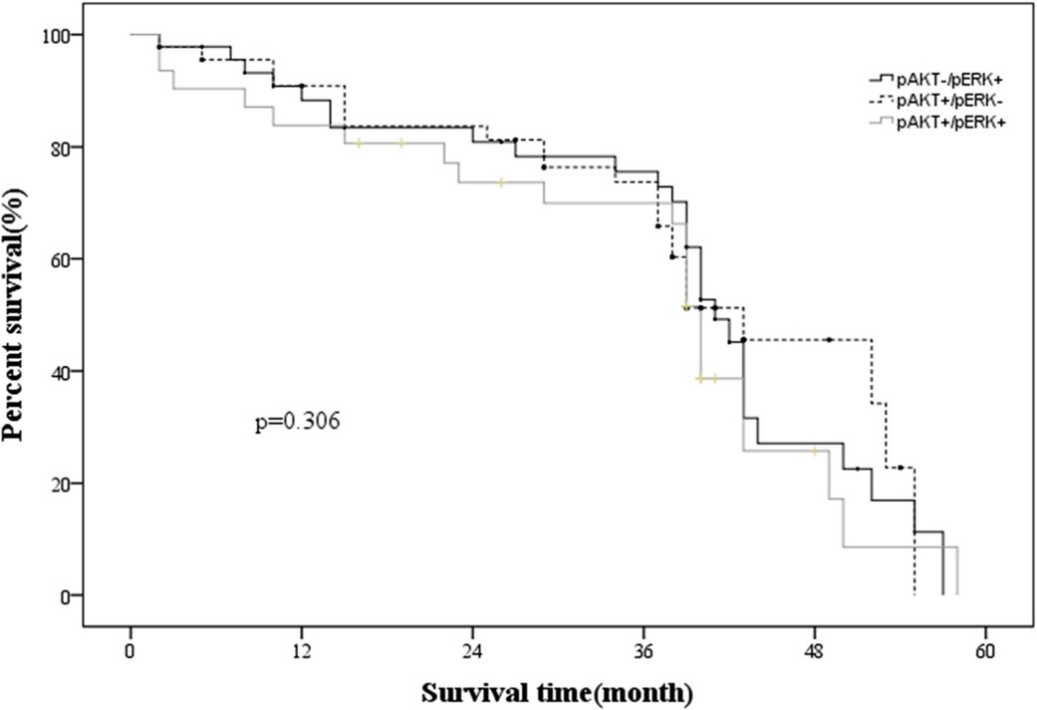
Comparison of Kaplan-Meier survival curves for patients with different p-AKT and p-ERK expression levels in patients with PHLPP low expression. They are no differences in survival regardless of the expression of p-AKT and p-ERK in patients with low expression of PHLPP (*P* = 0.306)

There were no significant differences in OS among the PHLPP-negative & p-AKT- positive, PHLPP-negative & p-ERK-positive and PHLPP-negative& p-AKT/p-ERK-positive (*p* = 0.306, Fig. 4).

## Discussion

Lung cancer is the most common malignant disease in the world, and is the leading cause of cancer morbidity and mortality in China, including both cities and countryside [15]. Strong evidence is emerging in the basic science literature that Akt and ERK are two signal transduction proteins that play important roles in carcinogenesis and chemoresistance [16]. PHLPP represents a family of novel Ser/Thr protein phosphatases that have been identified to negatively regulate signaling pathways activated including PI3K/Akt [1] and RAF/RAS/ERK in cancer cells [2]. Controlling the balance of protein phosphorylation is one of the most important defense mechanisms provided by protein phosphatases to prevent aberrant hyperactivation of signaling in cells [17]. Our studies here focused on elucidating the tumor suppressor function of protein phosphatases, PHLPP, in lung adenocarcinoma. In this study, we found that the expression of PHLPP were decreased in 76.5 % of lung adenocarcinoma tissues, which is consistent with previously findings in colon cancer, prostate cancer, chronic lymphocytic leukemia. Additionally, we found PHLPP expression was significantly correlated with tumor differentiation and T stage in lung adenocarcinoma. The level of PHLPP1 expression was significant related to the tumor T stage, in hypopharyngeal squamous cell carcinoma as reported by Zhou et al.

AKT and ERK signaling pathways are two important signaling pathways in the lung cancer [18]. The two signaling pathways are also the downstream signaling molecules of epidermal growth factor receptor (EGFR) signaling, which is mainly related with tumor occurrence and development. They also play an important role in the tolerance of chemoradiotherapy in lung cancer [19]. A few studies which assessed the role of Akt phosphorylation in NSCLC demonstrated that there was a statistically significant difference in survival between p-Akt-positive and p-Akt-negative patients and this difference was independent of tumor stage [20]. Activation of the ERK1/2 pathway is involved in malignant transformation both in vitro and in vivo. And the detection of immunoreactivity for p-ERK in patients with NSCLC is associated with advanced and aggressive tumors [21]. These data also suggest that the analysis of ERK1/2 activation may be useful to identify a subgroup of patients with a poorer prognosis. In the current study, we found PHLPP was inverse correlated with the expression of p-Akt and/or p- ERK in human lung adenocarcinoma tissues. Our findings here are consistent with our previous reports that PHLPP negatively regulated signaling pathways activated including PI3K/Akt and RAF/RAS/ERK in different cancer cells [1, 2]. Moreover, in this study, for the first time we detected PHLPP expressions in human lung adenocarcinoma. Furthermore, we found that low expression of PHLPP in lung adenocarcinoma highly correlated with shorter survival, which is consistent with the recent report that [22] downregulation of PHLPP expression contributed to hypoxia-induced resistance to chemotherapy in colon cancer cells. This may be related with the differences of treatment outcomes. Wang et al. reported that the OS time and relapse-free survival (RFS) time in PHLPP1-positive patients were significantly longer than in PHLPP1 negative patients and PHLPP1 was an independent prognostic factor for OS and RFS of gastric cancer patient. Therefore, PHLPP may play an important role in the dual function of two signal pathways and would be better to inhibit the growth of tumor.

## Conclusions

In conclusion, our study suggests that high levels of PHLPP might reflect a less aggressive lung adenocarcinoma phenotype and predict better survival in patients with lung adenocarcinoma. PHLPP can be a potential prognostic marker to screen patients for favorable prognoses.

## Abbreviations

PHLPP: PH domain leucine-rich repeats protein phosphatase
IHC: Immunohistochemistry
ECOG: Eastern Cooperative Oncology Group
PBS: Phosphate-buffered saline
SPSS: Statistical package for the social science
AJCC: American Joint Committee on Cancer
OS: Overall survival
RFS: Relapse-free survival
EGFR: Epidermal growth factor receptor
